# Supplementary material for: Patient involvement in the biopsychosocial integrated primary care model: A qualitative study in three health districts of South Kivu, Democratic Republic of Congo
Source: PLOS Glob Public Health. 2025 Dec 2;5(12):e0005548. doi: 10.1371/journal.pgph.0005548 (PMC12671736; doi:10.1371/journal.pgph.0005548)
Supplement: S1 Text — (DOCX) [file pgph.0005548.s001.docx]

Interview guide “**Patient involvement in the biopsychosocial integrated primary care model: A qualitative study in three health districts of South Kivu, Democratic Republic of Congo.”**

1. How do you find the relationship between you, the patients, and the caregivers? The provider sees you as a partner

2. Do you feel that the care offered respects your needs, preferences and independence?

3. Do you participate autonomously in decision-making about care that affects your life?

4. Do you discuss treatment options with the provider? Do you have a choice of treatment options? Does the provider guide you in choosing the best option for you, according to your needs? In addition to medication, what other treatment do you receive?

5. The patient has an important role to play and a great responsibility in person-centered care. How do you feel about this?

6. The patient's family plays an important role in person-centered care. How do you feel about this?

7. Do you discuss your care with support groups (family, patient associations)? Do these support groups have the time to discuss your care with your caregivers? Are support groups involved in planning at the health center?

8. Do providers discuss preventive measures and promotional activities with you in relation to your state of health? What preventive and promotional activities do you carry out in collaboration with providers and the community (support groups)?

9. Do providers help you make the connection between your health and other factors such as education, employment, family problems, etc.?

10. Do you think these support groups add value to this holistic care approach? Do you feel you receive all the information you need to improve your health?

11. Do providers and community health workers discuss with you how to live with your disease? Does this information help you to flourish in society?

12. What would you suggest to improve this biopsychosocial care, as part of person-centered care?
