# Supplementary material for: Patient involvement in the biopsychosocial integrated primary care model: A qualitative study in three health districts of South Kivu, Democratic Republic of Congo
Source: PLOS Glob Public Health. 2025 Dec 2;5(12):e0005548. doi: 10.1371/journal.pgph.0005548 (PMC12671736; doi:10.1371/journal.pgph.0005548)
Supplement: S1 Table — (DOCX) [file pgph.0005548.s002.docx]

**Summary Table of Standards for Reporting Qualitative Research (SRQR) as proposed by O'Brien et al.**

| **No.** | **Topic** | **Item** | **Line No in manuscript** |
| --- | --- | --- | --- |
|  | **Title and abstract** | | |
| S1 | Title | Concise description of the nature and topic of the study Identifying the study as qualitative or indicating the approach (e.g., ethnography, grounded theory) or data collection methods (e.g., interview, focus group) is recommended | 1 to 3 |
| S2 | Abstract | Summary of key elements of the study using the abstract format of the intended publication; typically includes background, purpose, methods, results, and conclusions | 28 to 55 |
|  | **Introduction** | | |
| S3 | Problem formulation | Description and significance of the problem/phenomenon studied; review of relevant theory and empirical work; problem statement | 57 to 166 |
| S4 | Purpose or research paradigm | Purpose of the study and specific objectives or questions | 117 to 118 |
|  | **Methods** | | |
| S5 | Qualitative approach and research paradigm | Qualitative approach (e.g., ethnography, grounded theory, case study, phenomenology, narrative research) and guiding theory if appropriate; identifying the research paradigm (e.g., postpositivist, constructivist/interpretivist) is also recommended; rationale | 203 to 209 |
| S6 | Researcher characteristics and reflexivity | Researchers’ characteristics that may influence the research, including personal attributes, qualifications/experience, relationship with participants, assumptions, and/or presuppositions; potential or actual interaction between researchers’ characteristics and the research questions, approach, methods, results, and/or transferability | 338 to 346 |
| S7 | Context | Setting/site and salient contextual factors; rationale | 121 to 201 |
| S8 | Sampling strategy | How and why research participants, documents, or events were selected; criteria for deciding when no further sampling was necessary (e.g., sampling saturation); rationale | 211 to 229 |
| S9 | Ethical issues pertaining to human subjects | Documentation of approval by an appropriate ethics review boardand participant consent, or explanation for lack thereof; otherconfidentiality and data security issues | 348 to 359 |
| S10 | Data collection methods | Types of data collected; details of data collection procedures including (as appropriate) start and stop dates of data collection and analysis, iterative process, triangulation of sources/methods, and modification of procedures in response to evolving study findings; rationale | 231 to 244 and 261 to 290 |
| S11 | Data collection instruments and technologies | Description of instruments (e.g., interview guides, questionnaires) and devices (e.g., audio recorders) used for data collection; if/how the instrument(s) changed over the course of the study | 245 to 261 and 286 to 290 |
| S12 | Units of study | Number and relevant characteristics of participants, documents, or events included in the study; level of participation (could be reported in results) | 362 to 372 |
| S13 | Data processing | Methods for processing data prior to and during analysis, including transcription, data entry, data management and security, verification of data integrity, data coding, and anonymization/deidentification of excerpts | 292 to 302 |
| S14 | Data analysis | Process by which inferences, themes, etc., were identified and developed, including the researchers involved in data analysis; usually references a specific paradigm or approach; rationale | 303 to 320 |
| S15 | Techniques to enhance trustworthiness | Techniques to enhance trustworthiness and credibility of data analysis (e.g., member checking, audit trail, triangulation); rationale | 321 to 337 |
|  | **Results/findings** | | |
| S16 | Synthesis and interpretation | Main findings (e.g., interpretations, inferences, and themes); might include development of a theory or model, or integration with prior research or theory | 374 to 385 |
| S17 | Links to empirical data | Evidence (e.g., quotes, field notes, text excerpts, photographs) to substantiate analytic findings | 389 to 605 |
|  | **Discussion** | | |
| S18 | Integration with prior work, implications, transferability, and contibution(s) to the field | Short summary of main findings; explanation of how findings and conclusions connect to, support, elaborate on, or challengeconclusions of earlier scholarship; discussion of scope of application/generalizability; identification of unique contribution(s) to scholarship in a discipline or field | 607 to 754 |
| S19 | Limitations | Trustworthiness and limitations of findings | 756 to 775 |
|  | **Others** |  |  |
| S20 | Conflicts of interest | Potential sources of influence or perceived influence on study conduct and conclusions; how these were managed | --- |
| S21 | Funding | Sources of funding and other support; role of funders in data collection, interpretation, and reporting | --- |
