# Supplementary material for: Patient involvement in the biopsychosocial integrated primary care model: A qualitative study in three health districts of South Kivu, Democratic Republic of Congo
Source: PLOS Glob Public Health. 2025 Dec 2;5(12):e0005548. doi: 10.1371/journal.pgph.0005548 (PMC12671736; doi:10.1371/journal.pgph.0005548)
Supplement: Attachment — Submitted filename: REVIEWER.pdf [file pgph.0005548.s006.pdf]

REVIEWER'S COMMENTS

Modification Note:

- **Line 143:**
  For the sake of uniformity, **Utilization**, **Curative**, and **Service** should begin with capital letters.

  Table 1. Description of health districts concerned by the study

Health District	Population (inhabitants)	Health Center Number	Hospital	Rates of utilization of curative services (%)*
Bagira (urban)	162,186	8	3	32.8
Katana (rural)	267,504	20	4	72.1
Walungu (rural)	318,610	23	3	32.5

  Legend: *(New Cases/Total Population) x 100. The health standard for the utilization rate of curative services is ≥50%. *(Source: health pyramid of South Kivu and District Health Information Software – DHIS-2, 2023)* (35)

- **Line 154:**
  The citation should read *KL et al. and Harrison SR & Jordan AM (36,37)* — not *KL et al., and Harrison SR & Jordan AM (36,37)*.
  The comma after "et al." should be removed. The correct form is *et al.* with a period, but **no comma**.

- **Line 157:**
  The correct in-text citation in Vancouver style is *Carman KL et al.* when citing multiple authors.

- **Figure 1 Caption:**
  Please remove the extra right parenthesis at the end of the caption:
  "Fig 1. Conceptual Framework for Patient Involvement in the BPS Model (inspired and adapted from Carman KL et al. and Harrison SR & Jordan AM (36,37))"
