# Supplementary material for: Patient involvement in the biopsychosocial integrated primary care model: A qualitative study in three health districts of South Kivu, Democratic Republic of Congo
Source: PLOS Glob Public Health. 2025 Dec 2;5(12):e0005548. doi: 10.1371/journal.pgph.0005548 (PMC12671736; doi:10.1371/journal.pgph.0005548)
Supplement: Attachment — Submitted filename: REVIEWER.docx [file pgph.0005548.s003.docx]

Reviewer’s Comments

Lines 54 and 55: Check the journal guidelines if keywords should be in alphabetical order.

Lines 97, 98 and 99: However, to our knowledge, patient involvement in the BPS model of care is poorly documented in the DRC.

This study explores patients' involvement in choosing their care strategies, their responsibility for holistic care, and their ability to support this BPS model.

I would have preferred the sentences to go like this:

However, to our knowledge, patient involvement in the BPS model of care is poorly documented in the DRC.

Therefore, this study explores patients' involvement in choosing their care strategies, their responsibility for holistic care, and their ability to support this BPS model.

**Between the two options, the second sentence is clearer and more coherent because of the logical transition provided by “Therefore,” which signals cause and effect more effectively.**

Line 117: Our study takes place at the operational level, which is comprised of HDs.

If the study has been completed then the sentence should be: Our study **took** place at the operational level, which is comprised of HDs.

Line 130: Could you provide a brief explanation of the concept of "Louvain Coopération"?

Line 134: You have to make the headings uniform. So, correct the heading in column 5, which should be Rates of Utilization of Curative Services

Similarly. In column two, use comma to indicate if the population is in thousands. For example, 162,186

Also, is it possible to have the data source in the reference list?

For example, source: *DHIS-2 (2023)* *Health pyramid of South Kivu and District Health*

*Information Software (29)*

Line 255: is ICA an abbreviation? If so, what is the full meaning.
