# Supplementary material for: Patient involvement in the biopsychosocial integrated primary care model: A qualitative study in three health districts of South Kivu, Democratic Republic of Congo
Source: PLOS Glob Public Health. 2025 Dec 2;5(12):e0005548. doi: 10.1371/journal.pgph.0005548 (PMC12671736; doi:10.1371/journal.pgph.0005548)
Supplement: Attachment — Submitted filename: Response to Reviewers.docx [file pgph.0005548.s005.docx]

To The Editor in chief	Bukavu, June 15th, 2025

PLOS GLOBAL PUBLIC HEALTH

Submission ID: PGPH-D-25-00933 - [EMID:65c5e292f74337b2]

Re: **Responses to Reviewers**

Dear Editor-In-Chief,

We are very pleased to submit the revised version of our manuscript entitled ***“*Patient involvement in the biopsychosocial integrated primary care model: A qualitative study in three health districts of South Kivu, Democratic Republic of Congo.*”***

We would like first to thank the Editor and Reviewers for their constructive comments. We have made the changes as recommended, by integrating suggested corrections and providing requested clarifications and answers to the questions raised.

We hope that we adequately addressed issues raised by the Reviewer and that the quality of the manuscript is improved.

Below is an itemized list of changes made to the revised version of the manuscript and point-by-point replies to the specific comments made by the Reviewer.

**ANSWERS TO JOURNAL REQUIREMENTS**

***Editor’s point #1:***

In the online submission form, you indicated that All data used and analyzed in this study are available from the corresponding author on reasonable request.

All PLOS journals now require all data underlying the findings described in their manuscript to be freely available to other researchers, either 
1. In a public repository, 
2. Within the manuscript itself, or 
3. Uploaded as supplementary information.

This policy applies to all data except where public deposition would breach compliance with the protocol approved by your research ethics board. If your data cannot be made publicly available for ethical or legal reasons (e.g., public availability would compromise patient privacy), please explain your reasons by return email and your exemption request will be escalated to the editor for approval. Your exemption request will be handled independently and will not hold up the peer review process, but will need to be resolved should your manuscript be accepted for publication. One of the Editorial team will then be in touch if there are any issues.

***Answer to Editor’s point #1:***

Thank you for your comment. We apologize for not being clear about data availability.

The data contains potentially sensitive information about the participants. Participants have been assured that "The information we collect during this research will be faithfully transcribed and stored in a computer file. Only the research team will have access to these files. The files will be secure (kept under lock and key or with a password)" and that their data will therefore not be made public. This approach has been approved by the Ethics Committee of the Catholic University of Bukavu, which authorized this research (UCB/CIES/NC/002/2024). Inquiries about the data can be addressed to [bkas2504@gmail.com](mailto:bkas2504@gmail.com) and to the Ethics Committee of the Catholic University of Bukavu ([vumilia.nakabanda@ucbukavu.ac.cd](mailto:vumilia.nakabanda@ucbukavu.ac.cd)).

***Editor’s point #2:***

Please provide separate figure files in .tif or .eps format.

***Answer to Editor’s point #2:***

Thank you for your requirement. We have taken it into consideration (.tiff format).

**ANSWERS TO REVIEWER 1**

***Reviewer’s point #1****:*

The authors go through the attached file and make the necessary corrections. In addition to this, the authors have done an impressive job in their research. As a concept, the biopsychosocial integrated primary care model can support vulnerable communities and enhance health and well-being for people.

***Answer to Reviewer’s point #1:***

Thank you very much for your general comment, which reassures us of the importance of the study.

***Reviewer’s point #2****:*

Lines 54 and 55: Check the journal guidelines if keywords should be in alphabetical order.

***Answer to Reviewer’s point #2:***

Thank you for your observation. According to the journal's guidelines, there is no requirement for keywords to be listed in alphabetical order.

***Reviewer’s point #3****:*

Lines 97, 98 and 99: However, to our knowledge, patient involvement in the BPS model of care is poorly documented in the DRC.

This study explores patients' involvement in choosing their care strategies, their responsibility for holistic care, and their ability to support this BPS model.

I would have preferred the sentences to go like this:

However, to our knowledge, patient involvement in the BPS model of care is poorly documented in the DRC.

Therefore, this study explores patients' involvement in choosing their care strategies, their responsibility for holistic care, and their ability to support this BPS model.

**Between the two options, the second sentence is clearer and more coherent because of the logical transition provided by “Therefore,” which signals cause and effect more effectively.**

***Answer to Reviewer’s point #3:***

Thank you very much for your suggestion. We adopted the second option, as you suggested to us (page 4, lines 107-108)

*Therefore, this study explores patients' involvement in choosing their care strategies, their responsibility for holistic care, and their ability to support this BPS model.*

***Reviewer’s point #4****:*

Line 117: Our study takes place at the operational level, which is comprised of HDs.

If the study has been completed then the sentence should be: Our study **took** place at the operational level, which is comprised of HDs.

***Answer to Reviewer’s point #4:***

Thank you very much for your comment. We have corrected the sentence (page 5, lines 120-121)

*Our study took place at the operational level, which is comprised of HDs.*

***Reviewer’s point #5****:*

Line 130: Could you provide a brief explanation of the concept of "Louvain Coopération"?

***Answer to Reviewer’s point #5:***

Thank you for your comment. Louvain Coopération is a Belgian academic non-governmental organization (NGO), created by the Université Catholique de Louvain (UCLouvain). It works in various fields, including health, food security, environmental protection and access to healthcare... In the DRC, particularly in the province of South Kivu, it supports the national program for non-communicable diseases in the management of certain chronic pathologies, notably diabetes, hypertension and mental health. We have provided a clarification in this regard (page 5, lines 136-139).

*The three HDs concerned by the study benefit from the support of “Louvain Coopération”, a Belgian academic non-governmental organization (NGO) of the “Université Catholique de Louvain” (UCLouvain), as part of the Non-Communicable Diseases Program (NCDP) from 2022 to 2026.*

***Reviewer’s point #6****:*

Line 134: You have to make the headings uniform. So, correct the heading in column 5, which should be Rates of Utilization of Curative Services

Similarly. In column two, use comma to indicate if the population is in thousands. For example, 162,186

Also, is it possible to have the data source in the reference list?

For example, source: *DHIS-2 (2023)* *Health pyramid of South Kivu and District Health*

*Information Software (29)*

***Answer to Reviewer’s point #6:***

Thank you for your helpful comment. We have reorganized the table as suggested and included the data source in the references (page 6, Table 1, and line 146)

*Table 1. Description of health districts concerned by the study*

| ***Health District*** | ***Population (inhabitants)*** | ***Health Center Number*** | ***Hospital*** | ***Rates of utilization of curative services (%)**** |
| --- | --- | --- | --- | --- |
| *Bagira (urban)* | *162,186* | *8* | *3* | *32.8* |
| *Katana (rural)* | *267,504* | *20* | *4* | *72.1* |
| *Walungu (rural)* | *318,610* | *23* | *3* | *32.5* |

And

*35. Ministry of Public Health. South Kivu Health Pyramid and District Health Information Software 2 (DHIS2). National Health Information System (SNIS) - DRC. 2023.*

***Reviewer’s point #7****:*

Line 255: is ICA an abbreviation? If so, what is the full meaning.

***Answer to Reviewer’s point #7:***

Thank you for your observation. We apologize for forgetting to mention it above. It's short for Inductive Content Analysis. We've made that clear (page 10, line 260)

*…* the Inductive Content Analysis***…***

**ANSWERS TO REVIEWER 2**

***Reviewer’s point #1****:*

Did the study have ethical approval - please add if so or address the issues of confidentiality, data storage etc.

***Answer to Reviewer’s point #1:***

Thank you very much for your comment. As mentioned in the ethical considerations (Ethics statement), the study was approved by the institutional health ethics committee of the Catholic University of Bukavu, under reference number UCB/CIES/NC/002/2024 (page 11, lines 288-289).

We have mentioned elements that enabled us to respect the confidentiality of respondents (page 8, lines 218-220).

*In all cases, these interviews occurred in an environment that ensured the confidentiality of the respondents' statements (in a room at the HC or a private area of the participant's residence).*

And (page 11, lines 294-295)

*To ensure confidentiality, respondents were guaranteed anonymity.*

We have just completed the elements relating to data storage and security (page 11, lines 295-296).

*The data were stored anonymously in the computer, with a password to ensure their protection and the confidentiality of the respondents.*

As they contain potentially sensitive information for the participants, access is only possible on request to the corresponding author and to the ethics committee of the Catholic University of Bukavu.

***Reviewer’s point #2****:*

**The intro and background could be more clear on what is the focus of the paper – and this should align more strongly with the conceptual framework that is used to analyse the interviews.**

- The paper moves between patient participation and community participation and between the concepts of PCC and quality care.
- Secondly, there are different descriptions of ‘involving people’ involving patients’ that make the introduction hard to follow.

I suggest clarifying the focus of the paper which I believe is on patient involvement in decision making for their care strategy aligned to the BPS model of care.

I would reread and make the description of the focus consistent throughout the paper Line 1: Involving people in providing care?

Line 85: involving patients in their care.

***Answer to Reviewer’s point #2:***

Thank you very much for your pertinent comments. We've enriched the introduction and corrected the confusion by referring to “patients” rather than “people”.

Please allow us to provide some clarification. Since the present work focuses not only on the patient-provider relationship or direct care but encompasses other aspects of care in a holistic manner, the word “patient” as used in this work is not limited to someone who just needs biomedical care. He lives in a sociocultural and psychosocial environment that influences him and other members of the community on a daily basis. This is why we prefer to understand this in the sense of person-centered care, and not patient-centered care, as Eklund mentions: *Håkansson Eklund J, Holmström IK, Kumlin T, Kaminsky E, Skoglund K, Höglander J, et al. “Same same or different?” A review of reviews of person-centered and patient-centered care. Patient Educ Couns [Internet]. 2019;102(1):3–11.*

And this links up with your concern to make the connection between the introduction and the conceptual framework we proposed for carrying out the study, the data collection which took place among people with chronic pathologies, both those who came for care to the Health Center, and those who were at home, stable from a somatic point of view. The aim was to gather their experiences in their daily lives, in managing their chronic diseases, and their involvement in decision-making in healthcare.

We have included an element that was missing from the introduction, the community (social support), to be more complete with the three components mentioned in this work, in connection with patient involvement: the individual himself, the healthcare provider and the community (social groups) (page 3, lines 78-81 and page 4, lines 88-90). This also enables us to be consistent with the results presented.

*Involving patients and their social supports has also shown its importance in improving the quality of their lives, in managing certain health conditions that require the BPS model of care, such as chronic pain management.*

***And***

*Some authors have even demonstrated patient satisfaction and adherence to treatment thanks to the support obtained from the social groups to which they belong.*

***Reviewer’s point #3****:*

Line 57-60: not only participation in health care policies – but also in implementation, monitoring of health services etc – 2024 WHA resolution 77.2 can be drawn on here also.

***Answer to Reviewer’s point #3:***

Thank you for your comment. We have completed the sentence according to your comment, and have taken into account the WHO resolution as proposed (page 3, line 61).

… as well as in implementing and monitoring health services

*6. World Health Organization (WHO). The Seventy-seventh World Health Assembly (WHA77.2): Social participation for universal health coverage, health and well-being. WHO Geneva [Internet]. 2024;1–5. Available from: https://apps.who.int/gb/ebwha/pdf_files/WHA77/A77_R2-en.pdf*

***Reviewer’s point #4****:*

Line 68 – suggest revision to explaining the benefit of patient and community involvement in decision making in health services .

***Answer to Reviewer’s point #4:***

Thank you very much for your very important suggestion. We've reworded the entire paragraph, instead indicating the factors that influence patient involvement in care and decision-making about their care (page 3, lines 69-72).

Various factors influencing patient involvement in care have been identified and explored worldwide. These include patients' health education and knowledge, their attitudes, beliefs, and values, their experiences of healthcare services, the provider-patient relationship (as partners in care), support groups…

But the following paragraph describes the benefits of involving patients in care (page 3, lines 73-81).

Some studies have shown the benefits of involving patients in decision-making about their healthcare. They have revealed that patients who are involved in decision-making in care express a sense of well-being and self-satisfaction. Others have mentioned improved interaction and communication between providers and patients, patient adherence to treatment, rapid adoption of healthy behaviors, and self-management of illness through active participation in self-monitoring activities. Involving patients and their social supports has also shown its importance in improving the quality of their lives, in managing certain health conditions that require the BPS model of care, such as chronic pain management.

***Reviewer’s point #5****:*

Line 72 – I think this sentence is not correct – not patient providing care- but patients involvement in decision making in care?

***Answer to Reviewer’s point #5:***

Thank you for your comment. It is indeed a mistake. We have corrected the sentence (page 3, lines 74-75).

They have revealed that patients who are involved in decision-making in care express a sense of well-being and self-satisfaction

***Reviewer’s point #6****:*

Line 74-5 – this example ‘considering patients as partners in care is better as part of the paragraph line 68-71

***Answer to Reviewer’s point #6:***

Thank you very much for your suggestion, which we have taken into consideration (page 3, lines 70-72).

These include patients' health education and knowledge, their attitudes, beliefs, and values, their experiences of healthcare services, the provider-patient relationship (as partners in care), support groups…

***Reviewer’s point #7****:*

**The basis for the BPS model is cited from 2 papers on diabetes**. That is not the original genesis of the model – it is better to include the original conceptualised more by George Engel (1977).

***Answer to Reviewer’s point #7:***

Thank you very much for your comment. We've taken it into account in the introduction (page 3, lines 64-65).

*One of the components of this PCC is the biopsychosocial (BPS) model of care, as proposed by George Engel in 1977, in which care is provided comprehensively (holistically), according to biological, psychological, social, and spiritual dimensions, while respecting the person's needs and values*

*7. Engel GL. The Need for a New Medical Model: A Challenge for Biomedicine. Science (80- ) [Internet]. 1977;196(4286):129–36. Available from: http://links.jstor.org/sici?sici=0036-8075%2819770408%293%3A196%3A4286%3C129%3ATNFANM%3E2.0.CO%3B2-F*

***Reviewer’s point #8****:*

**Defining quality of care:** Line 158- Quality care requires both qualitied personal and high-quality services – that sentence is not an oxymoron. If you want to define quality – then use a quote such as

*Quality* *Health care is health care delivery that is timely, efficient, effective, safe, people-centred, equitable (Institute of Medicine Committee on Quality of Health Care in, 2001)*

***Answer to Reviewer’s point #8:***

Thank you very much for your observation. We thought it best to delete this aspect of quality of care, so as not to deviate from the objective of our work.

***Reviewer’s point #9****:*

**Suggest to further make the direct link to patient engagement in their own care by drawing on a stronger Suggested frame for analysis**

For the conceptual frame used to analyse the interview – this could be better exemplified using an existing model – such as : Carmen 2013 ([Engaging patients to improve quality of care: a systematic review | Implementation Science | Full Text](https://implementationscience.biomedcentral.com/articles/10.1186/s13012-018-0784-z))

*Carman KL, Dardess P, Maurer M, Sofaer S, Adams K, Bechtel C, et al. Patient and family engagement: a framework for understanding the elements and developing interventions and policies. Health Aff. 2013;32(2):223–31.*

[Engaging patients to improve quality of care: a systematic review | Implementation Science | Full Text](https://implementationscience.biomedcentral.com/articles/10.1186/s13012-018-0784-z/figures/1)

**This paper is focussed on the green box** – on direct care – that is my understanding. And I think this is a useful conceptual model which provides clarity that we are not looking at patient engagement write large: [9789241511629-eng.pdf;sequence=1](https://iris.who.int/bitstream/handle/10665/252269/9789241511629-eng.pdf;sequence=1)

***Answer to Reviewer’s point #9:***

Thank you very much for your suggestion to improve our conceptual framework. We have enriched it, taking into account your observations, to be consistent with the results and the discussion. Relational aspects were thus developed, with emphasis on the patient-provider relationship, and above all, the patient-community relationship, a very important aspect in the management of chronic diseases.

Our framework aligns with Carman KL et al.'s definition of patient and family engagement: “patients, families, their representatives, and health professionals working in active partnership at various levels across the health care system—direct care, organizational design and governance, and policy making—to improve health and healthcare.” It is also in line with the other framework mentioned in the study (that of Harrison SR & Jordan AM), in the sub-Saharan African context. Given that our study focuses more on the first level of engagement according to the Carman KL et al. framework (Direct Care), we wanted to develop it further by exploring the three components in this engagement, in the implementation of the BPS model of care, also exploring the Harrison SR & Jordan AM framework. By providing these details, this proposed framework facilitates greater understanding of the interaction between these three components, in this partnership and the effective involvement of patients in care (pages ­6-7, lines 151-186).

*This study was based on the conceptual framework of patient involvement in the biopsychosocial model of care, which we developed by inspiring and adapting those of Carman KL et al., and Harrison SR & Jordan AM. This framework is represented in the following figure (Fig 1):*

*From a biopsychosocial perspective in direct care, patients' involvement in care and decision-making about their health depends on three interacting and mutually influencing components: the individual, the healthcare provider, and the community (social groups).*

- *Individual: The person is responsible for his or her health. In this context, “empowerment” (the process of acquiring power, which helps the individual or community to take control of their own lives, with the prospect of change and improvement) is a key element to be considered in the BPS model. The person is seen as an expert on his or her situation and body, an essential aspect in improving the quality of care. Successful treatment and recovery depend on participation, implication, and acceptance in care that the individual receives. Elements directly related to the individual are also considered, including biological and genetic characteristics, personal and social skills, lifestyle habits and behaviors, and socioeconomic characteristics.*
- *The healthcare provider: the interaction between patient and provider is crucial in this process of involvement in care decision-making. Empathy and compassion must characterize providers, in this partnership of care. At the front line of care, in the management of chronic diseases, a multidisciplinary approach should also be encouraged.*
- *Community (Social groups): Here, we have all the community groups and social networks (family, entourage, various associations or patient groups such as diabetic and hypertensive clubs), as well as community participation structures such as Health Area Development Committee, Community Animation Cell, Community-Based Organization, all of which act in immediate support of the individual, in collaboration with healthcare providers. The patient lives with other members of these social groups, in a sociocultural and psychosocial environment that constantly influences them. These elements need to be considered in care, especially in psychosocial support, a key component of the BPS model of care.*

*The form of the individual's involvement in decision-making and care according to the BPS model depends on the type of individual-care provider and individual-community relationship (from consultation to partnership and shared leadership), as shown in the figure (Fig. 1).*

***Reviewer’s point #10****:*

**Line 393: involvement in healthcare strategy – there is no example here of involvement in healthcare strategy .** This heading is not accurate – it implies healthcare plans and strategies. This is just about care decisions.

There are no examples of how the patient’s engage in monitoring care…the example mentions just how the club activities are progressing, and informing from CHW and Club president

***Answer to Reviewer’s point #10:***

Thank you very much for your comment. We have reworded the title to reflect the content mentioned (page 13, table 4, and page 16, line 397).

Involvement in decision-making and planning

***Reviewer’s point #11****:*

**Data collection using Interviews** – please add some details on ethical approval? How was confidentiality ensured, how was the data stored?

***Answer to Reviewer’s point #11:***

Thank you very much for your comment. As mentioned in the ethical considerations, the study was approved by the institutional health ethics committee of the Catholic University of Bukavu, under reference number UCB/CIES/NC/002/2024.

We have mentioned elements that enabled us to respect the confidentiality of respondents (page 8, lines 218-220 and page 11, lines 294-295).

*In all cases, these interviews occurred in an environment that ensured the confidentiality of the respondents' statements (in a room at the HC or a private area of the participant's residence).*

And

*To ensure confidentiality, respondents were guaranteed anonymity.*

We have just completed the elements relating to data storage and security (page 11, lines 295-296).

*The data were stored anonymously in the computer, with a password to ensure their protection and the confidentiality of the respondents.*

As they contain potentially sensitive information for the participants, access is only possible on request to the corresponding author and to the ethics committee of the Catholic University of Bukavu.

***Reviewer’s point #12****:*

Line 174 – need to spell out the acronyms.

***Answer to Reviewer’s point #12:***

Thank you for your comment. We have removed these abbreviations (page 7, lines 177-179).

*… such as Health Area Development Committee, Community Animation Cell, Community-Based Organization…*

***Reviewer’s point #13****:*

Line 127 – Community Animation Cell – this could benefit from a description.

***Answer to Reviewer’s point #13:***

Thank you very much for your suggestion. We have provided a description of the CACs for better understanding (page 5, lines 131-134).

*CHWs are grouped into Community Animation Cells (CACs) at the village level (a multi-sectoral structure of community participation at the peripheral level), and each CAC is represented at the HADC level by the CAC president.*

***Reviewer’s point #14****:*

Line 195 – need a more specific description of the club.

***Answer to Reviewer’s point #14:***

Thank you for your comment. We have reworded the sentence for better understanding (page 8, lines 198-201)

In these HAs, some patients organize themselves into clubs (diabetic and hypertensive clubs) in which various activities are organized for their care (health education, awareness-raising, exchanges of experience, mutual assistance within the framework of solidarity...).

***Reviewer’s point #15****:*

Line 257 – delete has

***Answer to Reviewer’s point #15:***

Thank you for your suggestion, which has been taken into account (page 10, lines 262-263).

The principal investigator began analyzing the data collected (BKM)

***Reviewer’s point #16****:*

Line 296 – around -can this not be precise?

***Answer to Reviewer’s point #16:***

Thank you very much for your comment. We have reworded the sentence for better understanding (page 11, lines 303-304)

… 45% were aged between 45 and 65.
