# Supplementary material for: Patient involvement in the biopsychosocial integrated primary care model: A qualitative study in three health districts of South Kivu, Democratic Republic of Congo
Source: PLOS Glob Public Health. 2025 Dec 2;5(12):e0005548. doi: 10.1371/journal.pgph.0005548 (PMC12671736; doi:10.1371/journal.pgph.0005548)
Supplement: Attachment — Submitted filename: Response_to_Reviewers_auresp_2.docx [file pgph.0005548.s007.docx]

To The Editor in chief	Bukavu, Jully 13th, 2025

PLOS GLOBAL PUBLIC HEALTH

Submission ID: PGPH-D-25-00933R1 - [EMID:ab84ca856dfc22f5]

Re: **Responses to Reviewers**

Dear Editor-In-Chief,

We are very pleased to submit the revised version of our manuscript entitled ***“*Patient involvement in the biopsychosocial integrated primary care model: A qualitative study in three health districts of South Kivu, Democratic Republic of Congo.*”***

First of all, we would like to thank the editor and reviewer for their comments to improve this manuscript. We have made the recommended changes, incorporating the suggested corrections.

We sincerely apologize for including the references suggested by the previous reviewer without warning you in advance. However, we had appreciated that they were relevant, unfortunately, we did not pay attention to the one that was retracted. We deeply apologize for this error. We have checked the references and removed the retracted and replaced with the correct.

We also used the PACE tool to analyse our figure. We were provided with the following results:

- Resolution was changed to 300 PPI
- Dimensions were adjusted to 7.49in W x 5.3in H
- TIFF file was converted to a valid TIF file

Below is an itemized list of changes made to the second revised version of the manuscript and point-by-point replies to the specific comments made by the Editor and Reviewer.

**ANSWERS TO JOURNAL REQUIREMENTS**

***Editor’s point #1:***

If the reviewer comments include a recommendation to cite specific previously published works, please review and evaluate these publications to determine whether they are relevant and should be cited. There is no requirement to cite these works unless the editor has indicated otherwise. 

Please review your reference list to ensure that it is complete and correct. If you have cited papers that have been retracted, please include the rationale for doing so in the manuscript text, or remove these references and replace them with relevant current references. Any changes to the reference list should be mentioned in the rebuttal letter that accompanies your revised manuscript. If you need to cite a retracted article, indicate the article’s retracted status in the References list and also include a citation and full reference for the retraction notice

***Answer to Editor’s point #1:***

Thank you for your comment. We sincerely apologize for including the references suggested by the previous reviewer without warning you in advance. However, we had appreciated that they were relevant, unfortunately, we did not pay attention to the one that was retracted. We deeply apologize for this error. We have checked the references and removed the retracted and replaced with the correct (see references).

**ANSWERS TO REVIEWER 1**

***Reviewer’s point #1****:*

Line 143:

For the sake of uniformity, **Utilization**, **Curative**, and **Service** should begin with capital letters.

Table 1. Description of health districts concerned by the study

| **Health District** | **Population (inhabitants)** | **Health Center Number** | **Hospital** | **Rates of utilization of curative services (%)*** |
| --- | --- | --- | --- | --- |
| Bagira (urban) | 162,186 | 8 | 3 | 32.8 |
| Katana (rural) | 267,504 | 20 | 4 | 72.1 |
| Walungu (rural) | 318,610 | 23 | 3 | 32.5 |

**Legend: *(New Cases/Total Population) x 100. The health standard for the utilization rate of curative services is ≥50%.** *(Source: health pyramid of South Kivu and District Health Information Software – DHIS-2, 2023)* (35)

***Answer to Reviewer’s point #1:***

Thank you for your observation. We've integrated capital letters at the beginning of words to respect uniformity, as suggested (page 6, table 1).

Table 1. Description of health districts concerned by the study

| **Health District** | **Population (inhabitants)** | **Health Center Number** | **Hospital** | **Rates of Utilization of Curative Services (%)*** |
| --- | --- | --- | --- | --- |
| Bagira (urban) | 162,186 | 8 | 3 | 32.8 |
| Katana (rural) | 267,504 | 20 | 4 | 72.1 |
| Walungu (rural) | 318,610 | 23 | 3 | 32.5 |

***Reviewer’s point #2****:*

**Line 154:** The citation should read *KL et al. and Harrison SR & Jordan AM (36,37)* — not *KL et al., and Harrison SR & Jordan AM (36,37)*. The comma after "et al." should be removed. The correct form is *et al.* with a period, but **no comma**.

***Answer to Reviewer’s point #2:***

Thank you for your comment. We have removed the comma (page 6, line 154)

… which we developed by inspiring and adapting from Carman KL et al. and Harrison SR & Jordan AM

***Reviewer’s point #3****:*

**Line 157:** The correct in-text citation in Vancouver style is *Carman KL et al.* when citing multiple authors.

***Answer to Reviewer’s point #3:***

Thank you very much for your suggestion, which we have taken into consideration (page 6, line 157)

… adapted from Carman KL et al. and Harrison SR & Jordan AM

***Reviewer’s point #4****:*

**Figure 1 Caption:** Please remove the extra right parenthesis at the end of the caption: *“Fig 1. Conceptual Framework for Patient Involvement in the BPS Model (inspired and adapted from Carman KL et al. and Harrison SR & Jordan AM (36,37))”*

***Answer to Reviewer’s point #4:***

Thank you very much for your observation. We have removed the parentheses (page 6, lines 156-157).

**Conceptual Framework for Patient Involvement in the BPS Model**, inspired and adapted from Carman KL et al. and Harrison SR & Jordan AM (36,37)
