# Supplementary material for: Patient involvement in the biopsychosocial integrated primary care model: A qualitative study in three health districts of South Kivu, Democratic Republic of Congo
Source: PLOS Glob Public Health. 2025 Dec 2;5(12):e0005548. doi: 10.1371/journal.pgph.0005548 (PMC12671736; doi:10.1371/journal.pgph.0005548)
Supplement: Attachment — Submitted filename: PLOS review Sept 2025.docx [file pgph.0005548.s008.docx]

**Generally**

An important topic on the relationship between patients and providers is highlighted by the authors. The paper is conceptualised robustly with an appropriate design. A final grammar check will be needed.

**Specifically**

Below are the specific feedback;

**Abstract**

Consideration between providers means what? Clarify this.

...mainly concentrate with the humanisation of care.

Five categories not clear, need to enlist them logically.

Any social economic status differences in participants.

Conclude in line with the five categories in the data.

**Introduction**

Involving patients ----line 57.

Add Social Ecological Model for involvement in care. Line 69 – 108. (individual – social network – community - societal).

Layers of patient behaviour determinants are more than social supports, that is, individual-social networks-community-societal.

More accurately, illustration of study location needed, use of a map is efficient. Line 47.

Add deeper provider attributes, that is, shared compassion, emotional arousal, aesthetic pleasure, simplification, asymmetry. Lines 171-174. Refer to Sadharanikaran model of communication:

Sadharanikaran Model of Communication

<https://sadharanikarantheory.blogspot.com/2017/11/sadharanikaran-model-of-communication.html#:~:text=Sadharanikaran%20model%20of%20communication%20(SMC,commonality%2C%20mutual%20understanding%20or%20oneness>. Accessed 02 Sept 2025

*Nandita Kapadia-Kundu, “Development and Application of Sadharanikaran Theory to Handwashing Behavior.”

**Data collection**

Bring out / mention any variations for participants met at HC and those met at home.

How were participants kept from sharing between themselves about study content? Were interviews held simultaneously for each HC? Mention how social desirability bias was controlled?

It is a coding process not a participant selection criteria that determines analysis. Lines 225-226. The second line 226 is not factually correct regarding analysis because as you select participants you don’t know diversity of information.

Lines 231-237 state why only two layers of the social ecological model were investigated in this study? The current investigation was about: patients and providers, patients and community, but did not include patients and social networks, patients and society, state why.

Social desirability bias introduced when an interpreter and an expert were part of the Shi language interview. How was bias managed? But later, you state there was only one interpreter. Which is which? Kindly be consistent.

Talk about the translation process clearly. Line 256.

What were the levels of coding? What were the condensed units? Line 264-274.

Were the final condensed units fitting the BPS model entirely? Were there no emergent condensed units? Can you share them incase they were in the data.

State the criteria for the total participants per health area? Move Table 2 to Results indicating planned and actual participants. Line 301-307. How many participants were interviewed at HC and how many were interviewed at homes?

**Discussion**

Implication needs to be stated per finding discussed. Then relate implication to relevant literature. Some of the literature is currently available.

**Limitations**

State differences between participants interviewed at HC and those interviewed at homes.
