# Supplementary material for: Patient involvement in the biopsychosocial integrated primary care model: A qualitative study in three health districts of South Kivu, Democratic Republic of Congo
Source: PLOS Glob Public Health. 2025 Dec 2;5(12):e0005548. doi: 10.1371/journal.pgph.0005548 (PMC12671736; doi:10.1371/journal.pgph.0005548)
Supplement: Attachment — Submitted filename: Response_to_Reviewers_auresp_3.docx [file pgph.0005548.s009.docx]

To The Editor in chief	Bukavu, Nov 8th, 2025

PLOS GLOBAL PUBLIC HEALTH

Submission ID: PGPH-D-25-00933R2 - [EMID:b237c22baf7d66c0]

Re: **Responses to Reviewers**

Dear Editor-In-Chief,

We are very pleased to submit the revised version of our manuscript entitled ***“*Patient involvement in the biopsychosocial integrated primary care model: A qualitative study in three health districts of South Kivu, Democratic Republic of Congo.*”***

First of all, we would like to thank the editor and reviewers for their comments to improve this manuscript. We have made the recommended changes, incorporating the suggested corrections.

As you will see in the manuscript, we have inserted additional works in the manuscript, including on the socio-ecological model, Sadharanikaran's communication model, and other work to enrich the discussion, at the request of the reviewers. We judged that their recommendation was necessary, which justified the addition of work relevant to our research topic.

Finally, we checked the references to comply with the journal's requirements.

Our revision includes

- The 'Response to Reviewers'.
- The 'Revised Manuscript with Track Changes'.
- The ‘Manuscript’

Below is an itemized list of changes made to the second revised version of the manuscript and point-by-point replies to the specific comments made by the Editor and Reviewers.

**ANSWERS TO JOURNAL REQUIREMENTS**

***Editor’s point #1:***

Please amend your online Financial Disclosure statement. If you did not receive any funding for this study, please simply state: “The authors received no specific funding for this work.”

***Answer to Editor’s point #1:***

Thank you for your comment. We have modified the Disclosure statement according to your orientation.

***Editor’s point #2:***

Please update your online Competing Interests statement. If you have no competing interests to declare, please state: “The authors have declared that no competing interests exist.”

***Answer to Editor’s point #2:***

Thank you for your comment. We have modified the Competing Interests statement according to your orientation.

***Editor’s point #3:***

If the reviewer comments include a recommendation to cite specific previously published works, please review and evaluate these publications to determine whether they are relevant and should be cited. There is no requirement to cite these works unless the editor has indicated otherwise.

***Answer to Editor’s point #3:***

Thank you for your comment. As you will see in the manuscript, we have inserted additional works in the manuscript, including on the socio-ecological model, Sadharanikaran's communication model, and other work to enrich the discussion, at the request of the reviewers. We judged that their recommendation was necessary, which justified the addition of work relevant to our research topic. We sent you an e-mail on this issue on October 26, 2025, to inform you and ask for your opinion.

***Editor’s point #4:***

Please review your reference list to ensure that it is complete and correct. If you have cited papers that have been retracted, please include the rationale for doing so in the manuscript text, or remove these references and replace them with relevant current references. Any changes to the reference list should be mentioned in the rebuttal letter that accompanies your revised manuscript. If you need to cite a retracted article, indicate the article’s retracted status in the References list and also include a citation and full reference for the retraction notice

***Answer to Editor’s point #4:***

Thank you for your comment. we have checked the references to comply with the journal's requirements (see references).

**ANSWERS TO REVIEWER 1**

***Reviewer’s point #1****:*

Line 643: Please remove the extra two full stops after disease.

***Answer to Reviewer’s point #1:***

Thank you for your comment. Following another reviewer’s recommendation, we revised the Discussion structure. Accordingly, the sentence has been modified as follows (lines 698-701):

Healthcare providers actively participate in Club sessions, offering guidance on hygiene, diet, and preventive behaviors. These educational activities foster functional, interactive, and critical components of health literacy.

***Reviewer’s point #2****:*

Line 658: "That's" is unacceptable in the manuscript. Please correct it to "That is."

***Answer to Reviewer’s point #2:***

Thank you for your comment. This part of sentence was also revised when the Discussion section was reworded (lines 720-726).

The need for a more compassionate and patient-centered approach emerged as a key area for strengthening the BPS model. Some participants associated the indifference and lack of empathy shown by certain healthcare providers with psychological distress, dissatisfaction, and reduced trust in care (factors known to undermine therapeutic outcomes). This finding is consistent with evidence that compassion and empathy are central to patients’ well-being and perceived quality of care.

***Reviewer’s point #3****:*

References: I noticed some inconsistencies in the reference formatting, particularly with capitalization and style of article titles. Please ensure the entire reference list is revised to align with the journal’s guidelines. (let the references to be uniform).

This is a uniform referencing:
20. Rinaudo CM, Van de Velde M, Steyaert A, Mouraux A. Navigating the biopsychosocial landscape: A systematic review on the association between social support and chronic pain. PLoS One [Internet]. 2025;20(4):e0321750. Available from: <https://doi.org/10.1371/journal.pone.0321750>

21. Ankomah SE, Fusheini A, Ballard C, Kumah E, Gurung G, Derrett S. Patient-public engagement for health system improvement in sub-Saharan Africa: A systematic scoping review. BMC Health Serv Res. 2021;21(1047):1–16.

***Answer to Reviewer’s point #3:***

Thank you very much for your comment. We uniformed all references according to the journal’s guidelines (see references).

**ANSWERS TO REVIEWER 3**

***Reviewer’s point #1****:*

1. Data Availability: While your current data availability statement acknowledges ethical restrictions and provides a contact pathway, it falls short of PLOS’s strong expectation for open data. PLOS requires that all data underlying the findings be made fully available without restriction, with rare exceptions for ethical or legal reasons. To strengthen compliance and increase transparency, I recommend:
a) Preparing a minimal, de-identified dataset that does not compromise participant confidentiality. For qualitative research, this could include:
i) The final codebook used in analysis.
ii) A matrix of themes and subthemes with anonymized participant identifiers.
iii) Illustrative, de-identified excerpts supporting each theme.
b) Depositing this dataset in a recognized open repository (e.g., OSF, Zenodo, Figshare) and including the DOI or stable link in your Data Availability Statement.
c) Clarifying restrictions by explicitly stating that full interview transcripts cannot be shared due to confidentiality and ethics approval constraints, but that qualified researchers may request further access through the corresponding author or your ethics committee.

***Answer to Reviewer’s point #1:***

Thank you for your recommendation and guidance. We have taken your suggestions into account. We have created a link and put the data, anonymously, respecting confidentiality.

**Minimal dataset available on:** <https://doi.org/10.5281/zenodo.17557004>

***Reviewer’s point #2****:*

Methods clarity & rigor
a) Describe how saturation was assessed (who decided, when, what evidence).
b) Add a brief reflexivity note (research team backgrounds, positionality, steps taken to mitigate bias).
c) Expand on interpreter influence and mitigation (e.g., member-checking, coder checks on translated segments).

***Answer to Reviewer’s point #2:***

Thank you very much for your comment. We have provided more precision for saturation, reflexivity and transcription, as requested, by completing the elements that were already mentioned in the manuscript.

Saturation (lines 221-225)

Data saturation was assessed by the principal investigator (BMK) in consultation with the supervising researchers, once no new codes emerged during the last three interviews. The decision was made collectively after reviewing coded transcripts and confirming that subsequent interviews yielded repetitive information.

Research reflexivity (lines 334-342)

The research team consisted of public health researchers with expertise in health systems in the Democratic Republic of Congo and qualitative research methods. All members were affiliated with academic institutions in South Kivu and Lubumbashi, but none were involved in the care of the study participants. The team recognized that their professional backgrounds could potentially influence data interpretation; therefore, reflexivity was maintained throughout the study by organizing debriefing meetings after each phase of data collection, comparing interpretations, and triangulating perspectives during the analysis to minimize individual bias.

Interpreter (lines 288-298): To minimize interpretation bias (as asked by another reviewer), we used the services of two independent expert translators, such as mentioned in this paragraph:

During data processing, the translation process was conducted rigorously to ensure both linguistic and conceptual accuracy. The recorded interviews were transcribed verbatim into Word files and then translated into French by two independent translators to ensure fidelity to participants’ words. To minimize interpreter bias, the translated transcripts were reviewed and cross-checked by coders for consistency of meaning, and any discrepancies were discussed among translators and coders to preserve the accuracy of participants’ intended meanings. Member verification of transcripts was not undertaken, as some authors have questioned its reliability as a validation technique, suggesting that it may alter participants’ original accounts or introduce recall bias. Instead, credibility was strengthened through coder and supervisor triangulation, peer debriefing, and back-translation procedures to ensure the accuracy and trustworthiness of the interpreted data.

***Reviewer’s point #3****:*

Results transparency: Insert a few additional verbatim quotes per major theme/subtheme in Table 4 to show analytic grounding.

***Answer to Reviewer’s point #3:***

Thank you for your suggestion. To avoid overloading the table, and following the suggestions of other reviewers, we have presented a separate table illustrating the analysis process (lines 326–327, Table 2). Additional details are provided in the data analysis Excel file, accessible via the link we created..

| Categories | Subcategories | Codes | Definition | Verbatim |
| --- | --- | --- | --- | --- |
| Patient participation | Involvement in community activities | Patient clubs to support patient care | Peer groups providing psychosocial support, information and mutual help in managing the disease. | *Sometimes, the club President tells us that he went to the HC in the meeting, and the providers asked him how the club's activities were progressing. He told us that we must sensitize others to come to the club.* |
|  |  | Concerted organization of home visits | Collaborative coordination of home visits between providers, CHWs and patients. | *I am a CHW. We organize home visits with the HN, and often, I go with our club President to see the other members in their households, and there we talk about their health. We are also informed by the nurse who oversees accompanying us, and we participate in HC activities. For example, if there's a visitor, vaccination or construction activities…* |
|  |  | Sensitize among entourage to ensure follow-up patient’s care | Involvement of family and friends in patient care and follow-up | *... that's why we're doing everything we can to reinforce the home visits, to avoid these harmful comments that lead to the patient being discriminated against and feared. People will see that caregivers and other companions or patients approach the patient. With these home visits, we make the rest of the family aware of the patient's follow-up, with respect for appointments or diet, and above all taking the patient directly to the hospital if he or she shows signs of discomfort.* |

***Reviewer’s point #4****:*

Limitations: Explicitly discuss selection bias/transferability stemming from club-member inclusion and convenience sampling, and any implications for generalisability to non-club patients.

***Answer to Reviewer’s point #4:***

Thank you very much for your suggestion. We've covered it in the limits. However, we have inserted other elements for clarification (lines 753-756 and 759-761).

This approach may have introduced selection bias, as club members likely differ from non-members in motivation, engagement, and peer support. Consequently, their experiences may not fully reflect those of patients receiving care outside such programs.

And

However, the detailed and contextualized description of participants’ experiences supports the transferability of the findings to similar primary care settings in low-resource or fragile contexts.

***Reviewer’s point #5****:*

Language & copy-editing: Perform a light language polish for minor grammar/phrasing issues to meet “standard English” criterion

***Answer to Reviewer’s point #5:***

Thank you for your comment. We believe we have made efforts to improve the grammar to meet the “standard English” criterion.

***Reviewer’s point #6****:*

Confirm non-duplication: Add a one-sentence statement in the manuscript explicitly confirming that results have not been published elsewhere

***Answer to Reviewer’s point #6:***

Thank you for your suggestion. We have added this statement after the acknowledgements (lines 803-804).

**Non-duplication**

The results of this study have not been published elsewhere.

**ANSWERS TO REVIEWER 4**

***Reviewer’s point #1****:*

An important topic on the relationship between patients and providers is highlighted by the authors. The paper is conceptualised robustly with an appropriate design. A final grammar check will be needed.

***Answer to Reviewer’s point #1:***

Thank you very much for your general comment, which reassures us of the importance of the study. We feel that we have made every effort to improve our English, so that it meets the criteria of standard English.

***Reviewer’s point #2****:*

Consideration between providers means what? Clarify this.

***Answer to Reviewer’s point #2:***

Thank you for your question. It's about interaction and mutual understanding. We've used the right word (lines 39-40)

Patients' participation in care revealed collaborative rapport between them and providers

***Reviewer’s point #3****:*

...mainly concentrate with the humanisation of care.

***Answer to Reviewer’s point #3:***

Thank you for the correction. We have taken it into account (line 47)

… mainly concentrated on humanizing care…

***Reviewer’s point #4****:*

Five categories not clear, need to enlist them logically.

Any social economic status differences in participants.

***Answer to Reviewer’s point #4:***

Thank you for your comment. The five categories introduce the sentences in the results. It was suggested that we present them in this way to respect the word limit in the abstract (lines 39, 41, 43, 45 and 46).

***Reviewer’s point #5****:*

Conclude in line with the five categories in the data.

***Answer to Reviewer’s point #5:***

Thank you for your suggestion. We have taken it into account, rewording the conclusion according to your orientation (lines 49-53)

Healthcare systems should consider all five dimensions identified in this study when defining policies and integrating biopsychosocial care. Strengthening these elements across the individual, provider, and community levels can enhance holistic patient involvement, promote self-management, and improve the quality and accessibility of care for chronic disease management in resource-limited settings.

***Reviewer’s point #6****:*

Involving patients ----line 57.

***Answer to Reviewer’s point #6:***

Thanks for the correction. We have taken it into account (line 57)

Involving patients

***Reviewer’s point #7****:*

Add Social Ecological Model for involvement in care. Line 69 – 108. (individual – social network – community - societal).

Layers of patient behaviour determinants are more than social supports, that is, individual-social networks-community-societal.

***Answer to Reviewer’s point #7:***

Thank you for your comment. We have expanded the description, emphasizing these points in the introduction (lines 73-82).

In addition to the biopsychosocial perspective, patient involvement in care can also be interpreted through the Social Ecological Model (SEM), which highlights the multiple, interacting layers that shape health behavior and participation in care. According to this model, individual actions and decisions are influenced not only by personal attributes and immediate social networks (such as family and support groups) but also by broader community and societal contexts, including health system organization, cultural norms, and policy environments. Patient involvement and empowerment depend on factors operating across these interconnected levels (from the individual and interpersonal to the community and societal spheres) each contributing to the enabling or constraining conditions of the biopsychosocial model of care.

Mcleroy KR, Bibeau D, Steckler A, Glanz K. An ecological perspective on health promotion programs. Heal Educ Behav. 1988;15(4):351–77.

Golden SD, Earp JAL. Social ecological approaches to individuals and their contexts: Twenty years of health education & behavior health promotion interventions. Heal Educ Behav. 2012;39(3):364–72.

***Reviewer’s point #8****:*

More accurately, illustration of study location needed, use of a map is efficient. Line 47.

***Answer to Reviewer’s point #8:***

Thank you for your suggestion. We have added the health map of South Kivu province, highlighting the HD concerned by the study (lines 123-124, Fig 1).

Fig 1. The three study HD on the health map of South Kivu province.

***Reviewer’s point #9****:*

Add deeper provider attributes, that is, shared compassion, emotional arousal, aesthetic pleasure, simplification, asymmetry. Lines 171-174. Refer to Sadharanikaran model of communication:

Sadharanikaran Model of Communication

<https://sadharanikarantheory.blogspot.com/2017/11/sadharanikaran-model-of-communication.html#:~:text=Sadharanikaran%20model%20of%20communication%20(SMC,commonality%2C%20mutual%20understanding%20or%20oneness>. Accessed 02 Sept 2025

*Nandita Kapadia-Kundu, “Development and Application of Sadharanikaran Theory to Handwashing Behavior.”

***Answer to Reviewer’s point #9:***

Thank you for your suggestion. We have taken it into account, integrating the elements related to the Sadharanikaran Communication Model (lines 176-184)

Beyond clinical competence, providers’ communication should integrate deeper relational attributes such as shared compassion, emotional arousal, aesthetic pleasure, simplification, and asymmetry*,* which reinforce trust and mutual understanding. These elements align with the **Sadharaṇikaraṇ Model of Communication**, which emphasizes sahridayata (a shared sense of empathy and emotional connection between communicator and receiver). In health contexts, this model suggests that providers and patients co-create meaning through affective resonance, even within asymmetric relationships, by simplifying messages and fostering emotional harmony.

Adhikary NM. An introduction to sadharanikaran model of communication. Bodhi An Interdiscip J. 2009;3(1):69–91.

Khadka U. Exploring the sadharanikaran model of communication from a health communication perspective. Bodhi An Interdiscip J. 2024;10(2):79–90.

***Reviewer’s point #10****:*

Bring out / mention any variations for participants met at HC and those met at home.

How were participants kept from sharing between themselves about study content? Were interviews held simultaneously for each HC? Mention how social desirability bias was controlled?

***Answer to Reviewer’s point #10:***

Thank you for your suggestion. We added the elements that showed the variations for participants met at HC and those met at home (lines 257-260)

Participants interviewed at the HCs emphasized partnership and collaboration with providers. Those met at home focused more on family support, emotional experiences, and continuity of care. These differences reflected both institutional and household perspectives of patient involvement.

We have also expanded the paragraph on the conduct of interviews, mentioning the control of biases, including that of social desirability (lines 265-276)

Before beginning each interview, the researchers introduced themselves to participants and explained that the data were collected exclusively for research purposes. They emphasized voluntary participation and confidentiality to promote openness and trust. The interviewers maintained a neutral and empathetic attitude, listening carefully to participants’ experiences without guiding or influencing their responses. To reduce potential bias, interviews were conducted individually in private settings, and participants were reminded not to discuss the interview content with others until the end of data collection. The scheduling and locations of interviews were varied to minimize overlap between participants. As the interviewers were independent of the participants’ usual healthcare providers, this approach helped limit both information sharing and social desirability bias, while encouraging honest and spontaneous accounts of personal experiences.

***Reviewer’s point #11****:*

It is a coding process not a participant selection criteria that determines analysis (Lines 225-226). The second line 226 is not factually correct regarding analysis because as you select participants you don’t know diversity of information.

***Answer to Reviewer’s point #11:***

Thank you for this helpful clarification that we appreciate. We agree that the diversity of information is identified and handled during the coding and analysis process rather than determined at the participant selection stage. The sentence has been revised to accurately reflect this distinction (lines 238-240)

However, the qualitative method can face certain constraints, notably the diversity of information gathered during interviews, which can complicate data analysis. While participant selection followed predefined inclusion criteria to ensure relevance and variation in perspectives, thematic diversity was identified and managed during the coding and analysis process.

***Reviewer’s point #12****:*

Lines 231-237 state why only two layers of the social ecological model were investigated in this study? The current investigation was about: patients and providers, patients and community, but did not include patients and social networks, patients and society, state why.

***Answer to Reviewer’s point #12:***

We appreciate the reviewer’s insightful comment. In our framework, patient support groups were conceptualized as the social network level of the social ecological model, as they represent peer-based systems of mutual support and interaction. We have now clarified this point in the revised manuscript (lines 247-252). The societal and policy levels were not examined, as they were beyond the scope of the present study, which focused on patient, provider, and community interactions

In this study, we focused primarily on the interpersonal and community levels of the social ecological model, reflecting the relationships between patients and providers, and between patients and community-based support structures. The support groups examined in this context were considered to represent the social network level, as they function as peer-based systems of interaction and mutual assistance. Broader societal or policy-level factors were beyond the scope of this investigation.

***Reviewer’s point #13****:*

Social desirability bias introduced when an interpreter and an expert were part of the Shi language interview. How was bias managed? But later, you state there was only one interpreter. Which is which? Kindly be consistent. Talk about the translation process clearly. Line 256

***Answer to Reviewer’s point #13:***

Thank you very much for your comment. We clarified the interview process by being concise, using the same translator, and how we had minimized the social desirability bias (lines 277-286)

Interviews were conducted in Swahili or in the local language (Shi), according to respondents’ convenience. For interviews conducted in Shi, an experienced interpreter, who had no prior relationship with participants and was fluent in both languages, facilitated real-time translation. The same interpreter was used throughout to maintain consistency and minimize variation in interpretation. To reduce potential social desirability bias, the interpreter was trained to remain neutral and avoid influencing participants’ responses. Each interview lasted about 45 minutes and was audio-recorded. To verify translation fidelity and ensure accuracy of meaning, an independent language expert later reviewed the recordings against the interpreter’s translations. All audio files were then transferred to the computer for secure storage.

We have explained the transcription process, as also requested by another reviewer (lines 288-294)

During data processing, the translation process was conducted rigorously to ensure both linguistic and conceptual accuracy. The recorded interviews were transcribed verbatim into Word files and then translated into French by two independent translators to ensure fidelity to participants’ words. To minimize interpreter bias, the translated transcripts were reviewed and cross-checked by coders for consistency of meaning, and any discrepancies were discussed among translators and coders to preserve the accuracy of participants’ intended meanings.

***Reviewer’s point #14****:*

What were the levels of coding? What were the condensed units? Line 264-274.

Were the final condensed units fitting the BPS model entirely? Were there no emergent condensed units? Can you share them incase they were in the data.

***Answer to Reviewer’s point #14:***

Thank you very much for raising this important point. We confirm that the coding and category formation were conducted inductively. However, during interpretation, the resulting themes were organized in relation to the three components of our conceptual framework—the individual, healthcare provider, and social group—reflecting the Biopsychosocial (BPS) model. This clarification has been added to the revised Data Analysis section to highlight the distinction between the inductive coding process and the framework-guided interpretation (lines 299-316). We've even added a table illustrating the analysis process, as suggested by another reviewer

We analyzed the transcripts using the **Inductive Content Analysis** approach proposed by Vears and Gillam [56]. Given that the BPS model of care is little explored in the DRC, this approach was appropriate for capturing the lived experiences of patients with chronic diseases and generating practical insights to inform care and health policy [57]. The principal investigator (BKM) first familiarized himself with the data by reading and rereading the transcripts. The coding process occurred at three levels: (1) identifying meaning units from participants’ narratives and condensing them into concise formulations; (2) grouping these condensed units into codes and subcategories based on similarity; and (3) clustering subcategories into broader categories. To ensure credibility and trustworthiness, three other researchers (CENM, SLM, and GJM) joined the principal investigator in reviewing and validating the codes, verifying consistency with the data collected [58]. Their collaborative review led to the identification of additional emergent codes and the refinement of subcategories [59]. Although coding was conducted inductively, the interpretation of categories was subsequently organized according to the three components of our conceptual framework (the individual, the healthcare provider, and the social group) reflecting the Biopsychosocial (BPS) model. This approach allowed data-driven themes to emerge freely while situating them within a relevant conceptual framework that remains underexplored in the DRC context.

***Reviewer’s point #15****:*

State the criteria for the total participants per health area? Move Table 2 to Results indicating planned and actual participants. Line 301-307. How many participants were interviewed at HC and how many were interviewed at homes

***Answer to Reviewer’s point #15:***

Thank you for your concern and suggestion. We have clarified the selection criteria in the Study participant selection section. Specifically, we indicated that we predefined a sample of five participants per health area for reasons of feasibility (lines 207-208). We have moved Table 2 to the results, indicating planned and actual participants, as well as those interviewed at the health center (Table 3, lines 358-361).

A total of 27 patients took part in the study, compared with the planned number, as shown in the table below (Table 3). Of the 27 participants, 11 were interviewers at the HCs.

Table 3: **Persons interviewed based on origin (HA)**

| Health District | Health Area | Planned | Interviewed | Interviewed at Health Center |
| --- | --- | --- | --- | --- |
| Bagira | Lumu | 5 | 4 | 1 |
|  | Nyamuhinga | 5 | 5 | 2 |
| Katana | Birava | 5 | 5 | 1 |
|  | Kabushwa | 5 | 5 | 4 |
| Walungu | Bideka | 5 | 5 | 2 |
|  | Lurhala | 5 | 3 | 1 |
| Total |  | 30 | 27 | 11 |

***Reviewer’s point #16****:*

Implication needs to be stated per finding discussed. Then relate implication to relevant literature. Some of the literature is currently available

***Answer to Reviewer’s point #16:***

Thank you very much for your pertinent observation and suggestion. We have restructured the discussion as you recommended, also indicating the implication for each major finding, and we have enriched the literature as well (lines 603-758).

***Reviewer’s point #17****:*

State differences between participants interviewed at HC and those interviewed at homes.

***Answer to Reviewer’s point #17:***

Thank you for your suggestion. We have taken it into account and included it in the limits (lines 761-765)

The interview setting (HCs or participants’ homes) may have influenced the depth and tone of responses. Participants interviewed at HCs might have felt less comfortable expressing critical views toward providers, whereas those interviewed at home could share more freely about personal, family, or psychosocial experiences. Despite this variation, it enriched the diversity and authenticity of the data.
