# Supplementary material for: Patient involvement in the biopsychosocial integrated primary care model: A qualitative study in three health districts of South Kivu, Democratic Republic of Congo
Source: PLOS Glob Public Health. 2025 Dec 2;5(12):e0005548. doi: 10.1371/journal.pgph.0005548 (PMC12671736; doi:10.1371/journal.pgph.0005548)
Supplement: Attachment — Submitted filename: Reviewer comments May 26.docx [file pgph.0005548.s004.docx]

**Review comments**

**May 23 2025**

**I have 3 overall comments.**

1. **The intro and background could be more clear on what is the focus of the paper – and this should align more strongly with the conceptual framework that is used to analyse the interviews.**

- The paper moves between patient participation and community participation and between the concepts of PCC and quality care.
- Secondly, there are different descriptions of ‘involving people’ involving patients’ that make the introduction hard to follow.

I suggest clarifying the focus of the paper which I believe is on patient involvement in decision making for their care strategy aligned to the BPS model of care.

I would reread and make the description of the focus consistent throughout the paper Line 1: Involving people in providing care?

Line 85: involving patients in their care.

Line 57-60: not only participation in health care policies – but also in implementation, monitoring of health services etc – 2024 WHA resolution 77.2 can be drawn on here also.

Line 68 – suggest revision to explaining the benefit of patient and community involvement in decision making in health services .

Line 72 – I think this sentence is not correct – not patient providing care- but patients involvement in decision making in care?

Line 74-5 – this example ‘considering patients as partners in care is better as part of the paragraph line 68-71

2. **The basis for the BPS model is cited from 2 papers on diabetes**. That is not the original genesis of the model – it is better to include the original conceptualised more by George Engel (1977).
3. **Defining quality of care:** Line 158- Quality care requires both qualitied personal and high-quality services – that sentence is not an oxymoron. If you want to define quality – then use a quote such as

*Quality* *Health care is health care delivery that is timely, efficient, effective, safe, people-centred, equitable (Institute of Medicine Committee on Quality of Health Care in, 2001)*

**2. Suggest to further make the direct link to patient engagement in their own care by drawing on a stronger Suggested frame for analysis**

For the conceptual frame used to analyse the interview – this could be better exemplified using an existing model – such as : Carmen 2013 ([Engaging patients to improve quality of care: a systematic review | Implementation Science | Full Text](https://implementationscience.biomedcentral.com/articles/10.1186/s13012-018-0784-z))

*Carman KL, Dardess P, Maurer M, Sofaer S, Adams K, Bechtel C, et al. Patient and family engagement: a framework for understanding the elements and developing interventions and policies. Health Aff. 2013;32(2):223–31.*

[Engaging patients to improve quality of care: a systematic review | Implementation Science | Full Text](https://implementationscience.biomedcentral.com/articles/10.1186/s13012-018-0784-z/figures/1)

**This paper is focussed on the green box** – on direct care – that is my understanding. And I think this is a useful conceptual model which provides clarity that we are not looking at patient engagement write large: [9789241511629-eng.pdf;sequence=1](https://iris.who.int/bitstream/handle/10665/252269/9789241511629-eng.pdf;sequence=1)

**Line 393: involvement in healthcare strategy – there is no example here of involvement in healthcare strategy .** This heading is not accurate – it implies healthcare plans and strategies. This is just about care decisions.

- There are no examples of how the patient’s engage in monitoring care…the example mentions just how the club activities are progressing, and informing from CHW and Club president

4. **Data collection using Interviews** – please add some details on ethical approval? How was confidentiality ensured, how was the data stored?
5. **Language/ clarity of phrasing**

Line 174 – need to spell out the acronyms.

Line 127 – Community Animation Cell – this could benefit from a description.

Line 195 – need a more specific description of the club.

Line 257 – delete has

Line 296 – around -can this not be precise?
